# The Bacteriophage Carrier State of *Campylobacter jejuni* Features Changes in Host Non-coding RNAs and the Acquisition of New Host-derived CRISPR Spacer Sequences

**DOI:** 10.3389/fmicb.2016.00355

**Published:** 2016-03-23

**Authors:** Steven P. T. Hooton, Kelly J. Brathwaite, Ian F. Connerton

**Affiliations:** Division of Food Sciences, School of Biosciences, University of NottinghamLoughborough, UK

**Keywords:** CRISPR, ncRNA, campylobacter, bacteriophage, carrier state lifecycle, RNA-Seq

## Abstract

Incorporation of self-derived CRISPR DNA protospacers in *Campylobacter jejuni* PT14 occurs in the presence of bacteriophages encoding a CRISPR-like Cas4 protein. This phenomenon was evident in carrier state infections where both bacteriophages and host are maintained for seemingly indefinite periods as stable populations following serial passage. Carrier state cultures of *C. jejuni* PT14 have greater aerotolerance in nutrient limited conditions, and may have arisen as an evolutionary response to selective pressures imposed during periods in the extra-intestinal environment. A consequence of this is that bacteriophage and host remain associated and able to survive transition periods where the chances of replicative success are greatly diminished. The majority of the bacteriophage population do not commit to lytic infection, and conversely the bacterial population tolerates low-level bacteriophage replication. We recently examined the effects of *Campylobacter* bacteriophage/*C. jejuni* PT14 CRISPR spacer acquisition using deep sequencing strategies of DNA and RNA-Seq to analyze carrier state cultures. This approach identified *de novo* spacer acquisition in *C. jejuni* PT14 associated with Class III *Campylobacter* phages CP8/CP30A but spacer acquisition was oriented toward the capture of host DNA. In the absence of bacteriophage predation the CRISPR spacers in uninfected *C. jejuni* PT14 cultures remain unchanged. A distinct preference was observed for incorporation of self-derived protospacers into the third spacer position of the *C. jejuni* PT14 CRISPR array, with the first and second spacers remaining fixed. RNA-Seq also revealed the variation in the synthesis of non-coding RNAs with the potential to bind bacteriophage genes and/or transcript sequences.

## Introduction

The functional role of CRISPR-Cas systems in *Bacteria* and *Archaea* is recognized as providing the benefit of acquired immunity to the prokaryotic kingdom. In order to counter the constant threats posed by mobile genetic elements the CRISPR-Cas system has evolved to survey, acquire, adapt, and destroy invading DNA in a sequence-specific manner. The invading elements may be in the form of plasmids or bacteriophages, which if not dealt with in a timely fashion could lead to the demise of the infected cell/bacterial population. The major components found in all CRISPR-Cas systems are the CRISPR-associated (Cas) proteins, Cas1 DNA endonuclease and Cas2 RNA endonuclease (Koonin and Krupovic, 2015; Selle and Barrangou, 2015). These proteins operate during the acquisition stage to generate protospacers (captured DNA) which are subsequently incorporated into the CRISPR array. Recently, analysis of mobile genetic elements known as transposons revealed a possible evolutionary origin for Cas1 protein. Named casposons, these transposable genetic elements were found to possess Cas1 DNA endonuclease domains as well as hallmarks of eukaryotic DNA transposons (B family DNA polymerase genes and terminal inverted repeats). Integration and excision of the casposon is proposed to be mediated by the encoded Cas1 endonuclease domain in a similar manner to how CRISPR spacer acquisition occurs in prokaryotes (Krupovic et al., 2014). Transcription of integrated spacers leads to the generation of CRISPR RNAs (crRNAs) that serve as RNA guides for the interference step (Hochstrasser and Doudna, 2015). Various other proteins combine to produce the interference effect, and the presence or absence of these proteins has allowed the classification of CRISPR-Cas systems into Type IA-U, Type IIA-C, Type IIIA-D, Type IV, and Type V (Makarova et al., 2015).

**Figure 1:**
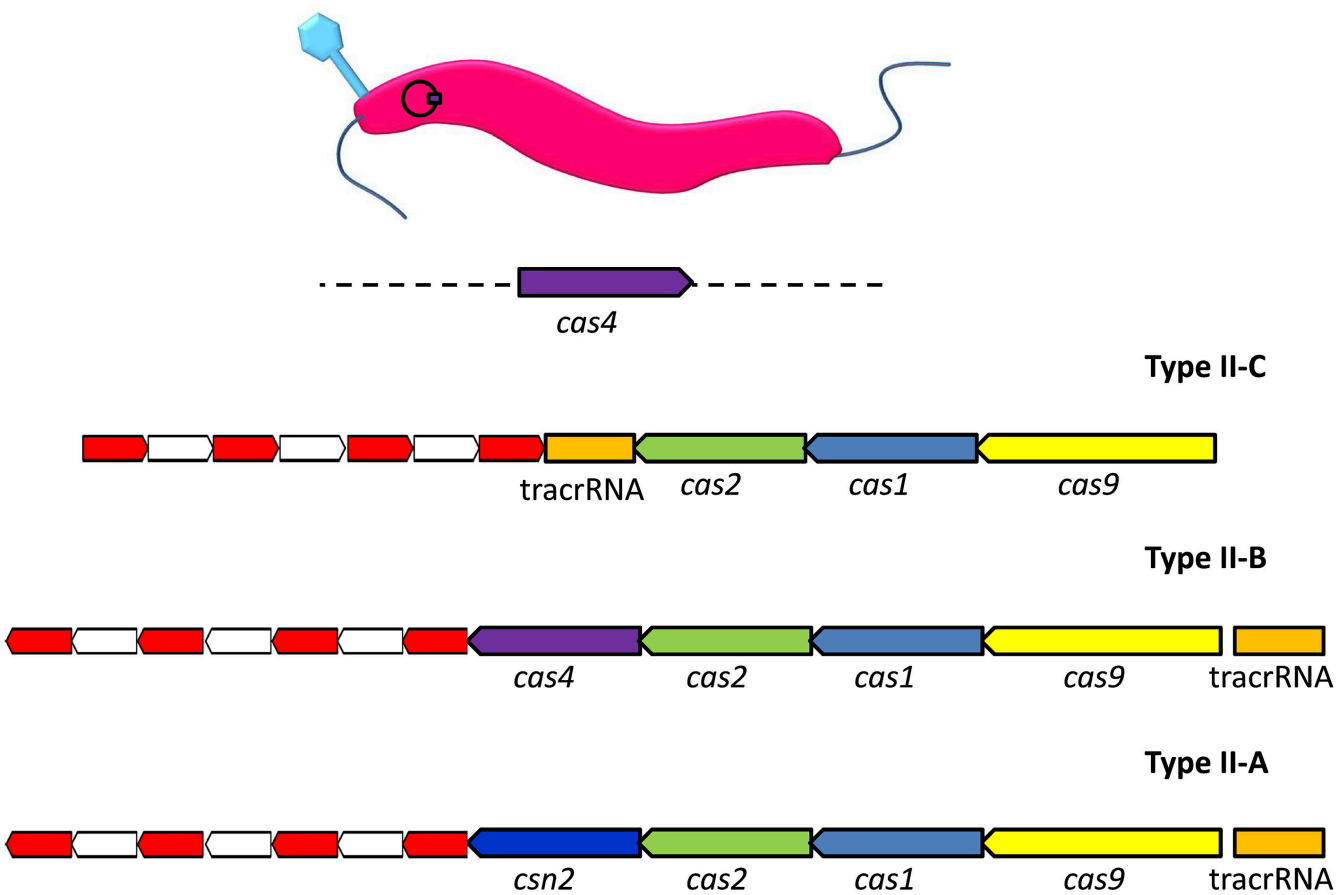
**Organization of the genomic loci of representative Type II CRISPR-Cas systems**. The type II-C arrangement represents the *Campylobacter jejuni* carrier state with the addition of bacteriophage encoded cas4 in *trans*. The red and white sections represent, respectively, direct repeats and spacer sequences. The analogous genes in the Type II systems are labeled and color coded, where Type II-B is *Legionella pneumophila* and Type II-A *Streptococcus thermophilus*.

Type II CRISPR-Cas systems have been shown to consist of several different permutations of genes encoding Cas1, Cas2, Csn2, Cas4, and Cas9 proteins (Figure 1). These proteins work in conjunction with trans-activated CRISPR RNAs (tracrRNAs), which are encoded within the sequence environment adjacent to the CRISPR array (Jiang and Doudna, 2015). The global gastrointestinal pathogen *Campylobacter jejuni* possesses a Type II-C CRISPR system. A recent survey of the available *C. jejuni* genome sequences reported that the Type II-C CRISPR system was almost universally present, with the exception that it was absent in representatives of the MLST clonal complex 42 (Pearson et al., 2015). Analysis of the distribution of CRISPR arrays in *C. jejuni* (*n* = 3746) and *Campylobacter coli* (*n* = 486) genome sequences indicated that CRISPR systems of *C. coli* strains isolated from agricultural environments were more closely related to those of *C. jejuni* rather than the CRISPR system encoded by *C. coli* isolates of non-agricultural origin. It is suggested that the shared environmental niche has driven the acquisition of a specific CRISPR system within and between circulating *C. jejuni* and *C. coli* (Pearson et al., 2015). Due to the almost ubiquitous distribution of CRISPRs and their associated genes within *C. jejuni* it may prove that these systems can be adopted as a means of typing different strains. However, for *C. coli*, the limited distribution of the CRISPR systems would prove to be a barrier to any successful CRISPR-based typing scheme (de Cárdenas et al., 2015).

By definition Type II-C CRISPR systems encode Cas1 and Cas2 nucleases for protospacer acquisition, tracrRNA which acts as a guide for targeting recognized foreign DNA, and Cas 9 protein which provides the immunity function (Chylinski et al., 2013; Plagens et al., 2015). The production of antisense CRISPR RNA requires the presence of host-encoded RNase III in Type II-C CRISPR systems. Subsequent reinfection events trigger the RNA-mediated targeting of Cas9 endonuclease to foreign DNA. This is facilitated by recognition of the dsRNA molecule that arises following complementary base pairing of tracrRNA to the 3′-end of the processed antisense crRNA (Chylinski et al., 2014).

The discovery of CRISPR-Cas systems has benefited molecular biology and the life sciences tremendously. Targeted genome editing utilizing CRISPR Cas9-based technologies has led to a revolution in functional genomics and how genes may be analyzed (Hsu et al., 2014). Ultimately the development of next generation technologies may well lead to major breakthroughs in our understanding of **gene expression** in health and disease (Sontheimer and Barrangou, 2015). The ability of Cas9 protein to introduce double-stranded breaks in target DNA sequences is a key component of CRISPR-based technologies. The precision afforded by Cas9-mediated DNA editing has provoked intense research in the fields of gene therapy (Barrangou, 2015). By combining Cas9 with a guide RNA, a chimeric RNA molecule consisting of tracrRNA:CRISPR RNA, it is possible to introduce breaks in dsDNA molecules in a highly-reproducible precise manner. DNA cleavage is achieved via the action of two Cas9 nuclease domains (HNH and RuvC-like), and with the aid of bespoke guide RNA transcripts it was found possible to target any given DNA sequence (Jinek et al., 2012).

However, functional studies of CRISPR-Cas systems in prokaryotes suggest there is a degree of complexity as to how they operate in nature. The evolutionary consequences of possessing CRISPR-Cas systems have required that both prokaryotes and phages adopt mechanisms capable of countering the potentially deleterious effects of CRISPR immunity. Recently it was shown that within a population of *S. epidermidis* RP62a, and in the presence of an invading plasmid, a sub-fraction of the culture was actively deleting its Type III-A CRISPR system. The obvious benefit to the mutant population is that it may acquire beneficial traits associated with the uptake of the plasmid. Conversely, the population which retains the system would survive plasmid-invasion by CRISPR-mediated immunity if uptake was proven to be deleterious (Jiang et al., 2013).

It is becoming evident that the evasion of CRISPR-mediated immunity is another key mechanism involved in the phage/bacteria co-evolutionary arms race that should be added to the established mechanisms including restriction–modification systems, alteration of outer membrane proteins countered by changes to adsorption apparatus, and abortive infection mechanisms (Plagens et al., 2015). For bacteriophages, the constant selective pressures imposed on their replicative success drive this evolutionary process, which has resulted in the emergence of a diverse array of anti-CRISPR mechanisms. Phage-encoded genes have the ability to inactivate the Type I-E and I-F CRISPR systems of *Pseudomonas aeruginosa* (Bondy-Denomy et al., 2013; Pawluk et al., 2014). The development of single nucleotide polymorphisms in protospacer adjacent motif (PAM) sequences prevents targeting of phage 2972 by the Type II-A CRISPR system of *Streptococcus thermophilus* DGCC7710 (Sun et al., 2013). In the presence of constant CRISPR-mediated selective pressure, phage 2972 was found to accumulate mutations in the genomic regions that were selectively targeted by the *S. thermophilus* DGCC7710 CRISPR system (Paez-Espino et al., 2015). *Myoviridae* phages which infect *Vibrio cholerae* have been found to encode a complete Type I-F CRISPR system. Acquisition of the system resulted in the ability to overcome *V. cholerae* anti-phage responses encoded within a pathogenicity island (Seed et al., 2013). As more CRISPR-Cas systems are studied within the *Bacteria* and *Archaea* it will be interesting to see what other resistance mechanisms emerge.

So far applications of CRISPR technologies and studies aimed at modifying lytic phages seem to be lacking behind the new-found roles for Cas9-technologies in genome engineering. Recently however an elegant study involving the above-mentioned *S. thermophilus* DGCC7710 and phage 2972 showed how Cas9-technologies can be used for the study of lytic phages (Martel and Moineau, 2014). The Cas9 protein encoded within the Type II-A CRISPR system of *S. thermophilus* DGCC7710 is already widely used in genome engineering (Jinek et al., 2012). Initially point-mutations were introduced into specific regions of the phage 2972 genome with an extremely high degree of efficiency. Introduction of a small 2 bp deletion in *orf39* led to a frameshift and successful inactivation of the target gene. Two further large deletions in *orf39* were also possible and as adjudged by smaller plaque sizes in phage 2972 *orf39* mutants it was concluded that this gene may be important for fitness of the phage. Of significant interest one of the main concepts to emerge from the study was the successful deletion of an open reading frame (*orf33*) previously shown to be dispensable, and its replacement with a bacterial gene. The gene introduced was the lactococcal methyltransferase *LladCHIA* which was found to be fully functional following insertion in phage 2972. It was observed that immunity to restriction via *in vivo* methylation of phage DNA was accomplished by phages bearing the introduced gene (Martel and Moineau, 2014). The prospect of efficient engineering of lytic phages capable of overcoming restriction-modification systems of any given host may be of exceptional use to industries such as phage therapeutics. Innate resistance to phages is often mediated via restriction–modification and alleviating this obstacle may allow advances to be made in trying to find novel phage-derived treatments for bacterial pathogens of humans, animals, and plants.

Aside from their classical function of providing a protective mechanistic response to invading genetic elements, non-canonical roles for CRISPR-Cas systems have been proposed. The ability to respond to invasive DNA elements such as plasmids and phage genomes has led to the discovery that stress responses triggered by disruption of membrane integrity can lead to activation of CRISPR-Cas systems (Ratner et al., 2015). Several important observations have delineated environmental signals that can be utilized by bacteria to regulate the expression of CRISPR-Cas systems. Transcriptional activation of CRISPR systems in *Salmonella enterica* and *Escherichia coli* can be mediated by the global DNA binding histone-like nucleoid structural protein (H-NS). Under normal circumstances H-NS represses CRISPR-Cas transcription; however in response to breaches in the integrity of the cell membrane H-NS becomes inactivated leading to transcription of the CRISPR-Cas system, and enabling the bacteria to respond to lethal infection or the acquisition of selfish genetic elements that can either compromise the microorganisms competitive ability or threaten the integrity of the genome (Westra et al., 2010; Medina-Aparicio et al., 2011).

Interestingly, other functional concepts have emerged from analyzing CRISPR-Cas biology. For the intracellular human pathogen *Francisella novicida*, the ability to survive exposure to potentially lethal insults such as antibiotics, membrane degradation, or perturbation, and host immunological responses is essential. Not only does *F. novicida* have to contend with cationic antimicrobial peptides, it is also required to have the ability to survive within the macrophage following phagocytosis wherein the bacterium needs to escape the phagosome in order to replicate in the macrophage cytosolic compartment. Intriguingly the Cas9 protein encoded within the Type II-B CRISPR system of *F. novicida* appears to be essential to overcome several barriers to successful completion of the intracellular lifecycle. These actions include resistance to the antibiotic polymyxin B (functionally akin to host cationic antimicrobial peptides), evasion of toll-like receptor 2 signaling and dampening of the inflammasome were all found to be reliant on the presence of a functioning Cas9 protein, tracrRNA and a scaRNA (Sampson et al., 2014).

## The *Campylobacter* bacteriophage carrier state

Analysis of bacteriophages that target *Campylobacter* spp. has led to many interesting insights into how they interact with their bacterial hosts. In some cases, what could be deemed as typical predator/prey behavior associated with lytic phages is foregone and is replaced by an equilibrium known as the carrier state. The nature of the carrier state is such that rather than committing to lysis the phage population is stably maintained in association with its host. Unlike temperate phages which commit to lysogeny and integrate their genomes into that of their host, *Campylobacter* phages remain associated as freely-replicating infective particles which propagate on a small fraction of the host population (Siringan et al., 2014; Brathwaite et al., 2015). This phenomenon has precedents in the literature with reports of **bacteriophage carrier states** being identified in *Shigella dysenteriae* (Li et al., 1961), *Brucella abortus* (Jones et al., 1962), *Proteus mirabilis* (Coetzee and Hawtree, 1962), mycobacteria (Baess, 1971), and most recently using phage P22 and *Salmonella typhimurium* LT2 (Cenens et al., 2013).

KEY CONCEPT 1 **Bacteriophage Carrier State**

The carrier state describes mixtures of bacteria and of bacteriophages that remain associated in a stable equilibrium. Carrier state cultures contain subpopulations of bacteria that either support bacteriophage replication or are resistant but can act as continuous founders of the sensitive subpopulation.

Analysis of carrier state cultures of *C. jejuni* PT14 confirmed that the phages and bacterial host remain associated in stable ratios even following serial passage representing multiple generations. Initial identification of the bacteriophage carrier state in *C. jejuni* PT14 arose following studies that were initiated to monitor the ability of Class III *Campylobacter* phages to disperse *Campylobacter* biofilms (Siringan et al., 2011). Bacteria recovered from the biofilms were found to spontaneously produce phage following disruption of the structure prompting further investigation of the origins of the phage burst. A combination of PFGE and Southern blot analysis was used in order to discount the unlikely event that otherwise virulent bacteriophage had formed lysogens by integration of *Campylobacter* phage genomic DNA. Non-integrated phage genomic DNAs (band size ~140 kb) were visible on PFGE gels containing *SmaI*-digested *C. jejuni* genomic DNA migrating alongside undigested *C. jejuni* DNA. Hybridization of Southern blots with *Campylobacter* phage specific probes for CP8 and CP30A confirmed that the 140 kb bands represented phage DNA and that the *SmaI*-fragments arising from bacterial chromosomal DNA contained no phage-hybridizing DNA sequences. This approach revealed that the phage genomes were present in carrier-state cultures as non-integrated genetic elements and absent from control cultures. *In vivo* studies showed that the carrier state bacteria were unable to colonize the poultry gut following analysis of the caecal contents of experimentally inoculated chickens (Siringan et al., 2014). Observations of planktonic growth phase-dependent parameters indicated that *C. jejuni* PT14 growing in the presence of bacteriophages CP8 and CP30A showed reduced fitness *in vitro*. Initially, bacteria growing during the early-logarithmic growth phase were found to be non-motile in carrier state cultures compared to non-carrier state cultures (uninfected controls). Coincident with the impairment in motility, bacteria isolated from early-logarithmic growth cultures displayed a vastly reduced ability to adhere to and infect HCA-7 colonic epithelial cells (Brathwaite et al., 2015). Transcriptional studies of the carrier state cultures revealed significant reduction in the expression of the *flaA* gene encoding the major flagellin protein of *C. jejuni*. Instead the carrier state cultures show increased σ54-dependent expression of the *flaB* gene, which encodes for a second normally minor flagellin protein (Brathwaite et al., 2015). Translation of the alternative flagellin genes results in proteins with similar amino acid sequences; it is therefore likely that the change in regulation is accompanied with other functional changes in the control of motility.

## *Campylobacter* phage Cas4 protein

Recently we reported the presence of a conserved Cas4-like ortholog in the genomes of both Class II and Class III *Campylobacter* phages (Hooton and Connerton, 2014). Cas4 proteins are reported as playing a role during the acquisition of protospacers in Type II-B CRISPR systems. The structural basis of Cas4 monomer is reliant on the presence of four conserved cysteines which together provide the basis for formation of a 4Fe-4S cluster. This structural arrangement often referred to as an “iron-staple” is exemplified in AddB nuclease of *Bacillus subtilis* and RecB nuclease of *E. coli*. A decameric toroidal configuration forms the quaternary structure of the protein which is observed to consist of five Cas4 dimers (Lemak et al., 2013). The *in vitro* activity of Cas4 protein isolated from the archaeon *Sulfolobus solfataricus* SSO0001 is reported to be a 5′–3′ single stranded DNA exonuclease. Specifically, the Cas4 protein was proposed to function in the generation of protospacer 3′ single stranded DNA ends that have the potential to undergo recombination with the CRISPR array (Zhang et al., 2012). Furthermore, the Cas4 protein was also shown to have the ability to perform ATP-independent DNA unwinding against single stranded DNA (Lemak et al., 2013). Recently, it was reported that a Cas4 protein isolated from the hyperthermophilic archaeon *Pyrobaculum calidifontis* contained a 2Fe-2S cluster (Lemak et al., 2014). In the case of 4Fe-4S Cas4 proteins the four conserved cysteines are absolutely required for maintaining structural integrity of the iron sulfur cluster and activity of the protein (Zhang et al., 2012). For 2Fe-2S Cas4, the nuclease function of the protein was found to remain intact following mutation of these residues, even though the iron sulfur cluster was lost (Lemak et al., 2014). Various nuclease domains/motifs are present in Cas4 proteins including RecB-like nuclease domains, PD(D/E)XK nuclease domain, and a QXXXY motif. *In silico* analysis of Cas4-like proteins in *Campylobacter* phages showed that all the domains and motifs described above are present. The conserved cysteine residues were found to be intact and even though very little amino acid homology is observed between intervening sequences in other *Campylobacter* phages, the core nuclease domains are also present. Analysis of phylogenetic trees drawn using Cas4 primary sequence data for several prokaryotes and *Campylobacter* phages highlighted the relatedness between their respective proteins. The addition of a phage-encoded Cas4 protein into the apparatus of the *C. jejuni* PT14 Type II-C CRISPR system (Cas1, Cas2, Cas9, and tracrRNA) creates a putative system that phenotypically resembles Type II-B CRISPR systems. Prior to analysis it could be hypothesized that if the carrier state was to function in a manner that allowed the survival of phages and their bacterial hosts then the host CRISPR system must be in some way inactive, or functioning in an atypical fashion. By combining the deep sequencing strategies of whole genome sequencing and RNA-Seq it was possible to identify rare molecular events which took place during carrier state associations between *C. jejuni* PT14 and *Campylobacter* phages CP8 and CP30A.

**Figure 2:**
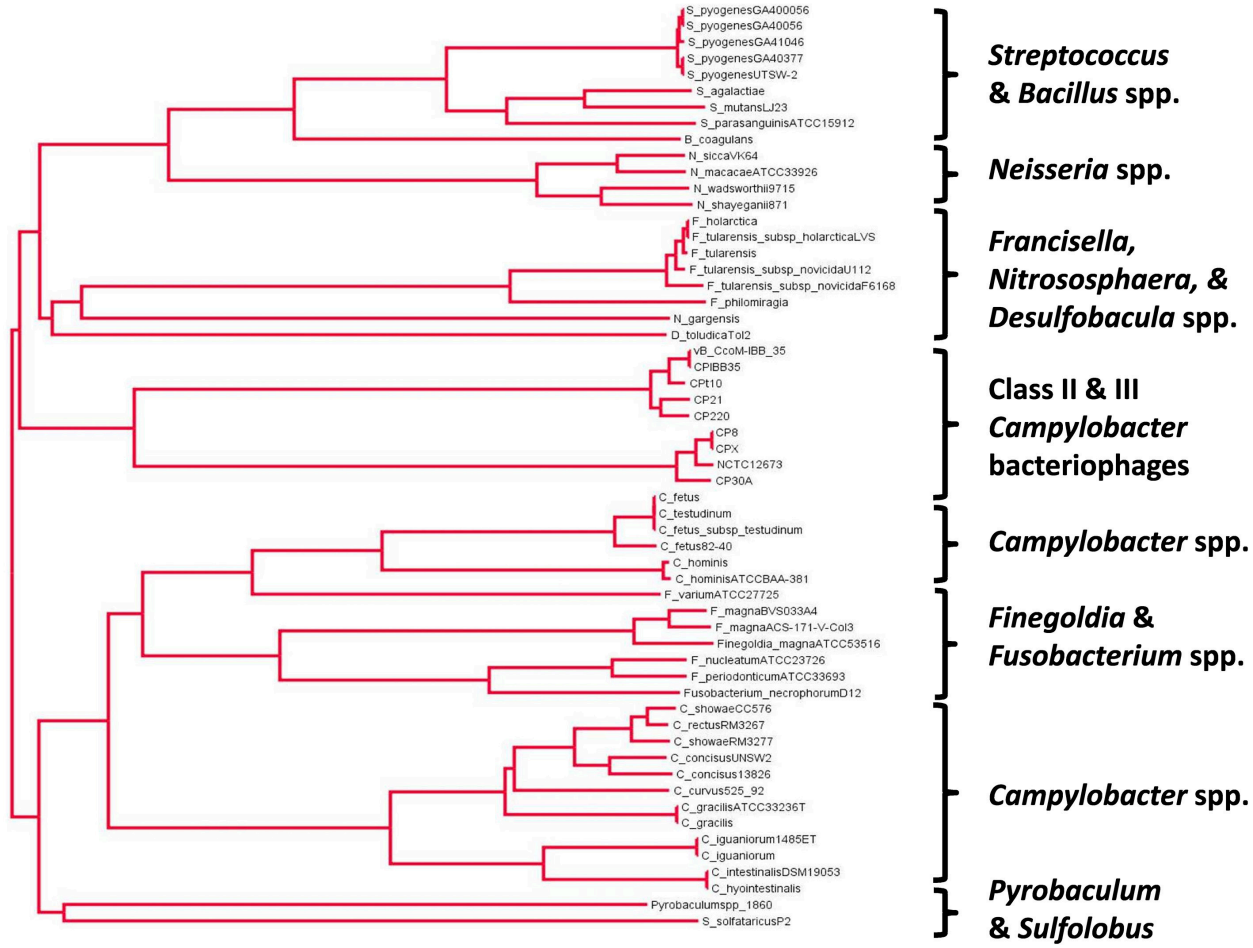
**Phylogenetic analysis of the Cas4 protein family related to the proteins encoded in class II and III *Campylobacter* bacteriophages using a neighbor-joining tree methodology (ClustalW2 alignment and FigTree v1.4.2.)**.

A phylogenetic tree drawn from the alignment of amino acid sequences of Class II and Class III *Campylobacter* phage Cas4-like proteins with a diverse selection of bacterial and archael Cas4 proteins is shown in Figure 2. The *Campylobacter* phages are found to cluster together based on their genome size such that Class II (170–190 kb) and Class III (110–150 kb) phages have their own branches. *C. coli* phages Cpt10 and vBCcoM-IBB_35 are also observed to cluster on their own sub-branch of the Class II lineage. A selection of *Campylobacter* spp. Cas4 proteins were included in the analysis in order to evaluate if the *Campylobacter* **phage-encoded Cas4** protein originated within the bacterial genus. Analysis of aligned amino acid sequences shows that conservation between phage and bacterial Cas4 protein appears restricted to the conserved cysteine residues and **nuclease** domains found within the proteins.

KEY CONCEPT 2 **Phage-encoded Cas4 nuclease**

Cas4 proteins have a functional role in the protospacer acquisition of Type II-B CRISPR systems. An analogous role for a phage-encoded Cas4 could confer a phage activated CRISPR immunity and/or autoimmunity in host bacteria with alternative Type II CRISPR systems.

### *C. jejuni* spacer acquisition

The unexpected finding of a Cas4-like protein in *Campylobacter* phages prompted us to analyze the CRISPR array of *C. jejuni* PT14 specifically during carrier state infections. Bioinformatic analysis of Cas4-like proteins of Class II and III *Campylobacter* phages showed they were conserved throughout, and the proteins all bore the hallmarks of Cas4 proteins. For the analysis two well characterized phages that were identified as encoding a Cas4-like protein (Class III *Campylobacter* phages CP8 and CP30A) were selected on the basis of their ability to establish carrier state cultures with *C. jejuni* PT14, and that the host CRISPR array did not already feature spacers directed at these phages (Siringan et al., 2014; Brathwaite et al., 2015). However, evidence suggests that it is possible to acquire spacer sequences directed at the genomes of the phages CP8 and CP30A since corresponding spacer DNA sequences have been noted in CRISPR arrays of *C. jejuni* genomes deposited in public databases (Pearson et al., 2015). Using these two phages it was possible to observe *de novo* **spacer acquisition** but the spacers consisted solely of host-derived sequences. In the absence of phages CP8 or CP30A no alterations were observed in the *C. jejuni* PT14 CRISPR array with the native **self-derived spacers** remaining fixed.

KEY CONCEPT 3 **Activated self-spacer acquisition**

If the CRISPR array can fix host spacer sequences then the interference mechanism can target the host genome itself resulting in cell death, a situation termed CRISPR-mediated autoimmunity.

To try to gain an understanding of the impact of carrier state infection upon *C. jejuni* PT14 an in-depth molecular analysis of the Type II-C CRISPR array was performed. Encoded upstream of the gene *moe2* (A911_07320) in *C. jejuni* PT14—Acc. No. CP003831 (Brathwaite et al., 2013) is a CRISPR array containing four 36 bp direct repeats with three intervening 30 bp spacer sequences. Analysis of the whole genome sequence of *C. jejuni* PT14 revealed that the 30 bp spacers found within the CRISPR array were representative of self-genes, rather than the expected plasmid or phage sequences. Intriguingly two of the spacers have homology with genes that are associated with externally presented proteins—peptidoglycan-associated lipoprotein Omp18 (18/30 nucleotides—A911_00540) and apolipoprotein N-acyltransferase (16/30 nucleotides—A911_05300). Analysis of RNA-Seq reads for these sequences indicated that apolipoprotein N-acyltransferase is transcribed at very low levels in *C. jejuni* PT14. This may allow the bacterium to evade immunological detection by toll like receptor 2 (TLR2) specifically by down-regulating the production of stimulatory acylated lipoproteins. Further spacer sequences were identified which matched the sequences of an isoleucyl tRNA synthetase (15/30 nucleotides—A911_05135), and a di/tripeptide transporter (12/30 nucleotides—A911_03195). The acquisition of spacer sequences of host-origin in carrier state cultures was found to be in stark contrast to the fixed nature of the *C. jejuni* PT14 CRISPR array. For CP8 carrier state cultures two of the spacers acquired showed complete matches to genes which are associated with motility and capsular polysaccharide biosynthesis. The motility-associated spacer was found to target the gene encoding the motility-associated protein PseE (30/30 nucleotides—A911_06495) which may have some bearing on the lack of motility observed in *C. jejuni* PT14 carrier state cultures. Loss of invasion and adherence capabilities of carrier state cultures may also be influenced by acquisition of the spacer targeting the capsular polysaccharide biosynthesis gene (30/30 nucleotides—A911_06909). A similar scenario was observed for CP30A carrier state cultures in which several different spacers were observed to target genes exclusively of host origin with perfect or near-perfect matches to the *C. jejuni* PT14 genome. The most notable acquisition was multiple spacers targeting the genes ADP-heptose-lipooligosaccharide-heptosyltransferase I and II.

The terminal direct repeat of the CRISPR array is positioned 144 bp upstream of the genes *cas2* (A911_07325), *cas1* (A911_07330), and *cas9* (A911_07335) which are accompanied by tracrRNA. The generation of pre-crRNAs occurs via transcription from a −10 transcriptional start site (5′-TAAAAAT-3′) located 116 bp upstream of the CRISPR array. Independent promoters with similarity to extended −10 transcriptional start sites (5′-GGTAAAAAT-3′) are also found preceding each direct repeat. A hallmark of Type II CRISPR systems is the co-ordination of endogenous RNase III/tracrRNA during processing to produce mature crRNAs during the biogenesis step (Deltcheva et al., 2011; Dugar et al., 2013) Transcriptomic analysis of early exponential phase *C. jejuni* PT14 carrier state cultures showed that several genes associated with RNA biogenesis and RNA processing are differentially upregulated including RNase III (Brathwaite et al., 2015).

### Small RNAs

Differences in the expression of non-coding RNAs were evident in the carrier state cultures compared to wild type *C. jejuni* PT14, most notably the accumulation of **sRNA**s. RNA-seq analysis identified sRNAs with heterogeneity at their termini implying they were generated by RNase processing or perhaps mis-processing. For example, PT14CP30 carrier state cultures contained an over-represented sRNA mapping to an intergenic genomic sequence between tRNAs (A911_t08346–A911_t08348), and exhibiting a 40/51 bp match to the CP30 phage genome. Association between complementary regions of bacterial and phage RNAs could lead to the preservation of the paired regions and exoribonuclease processing of the unpaired ends leading to the observed terminal heterogeneity. Other antisense sRNAs of chromosomal origin have the potential to bind phage transcripts (>25 nucleotide sequence matches) and act as *trans*-encoded inhibitory RNAs to suppress phage expression. Duplex RNA structures would also require cleavage by RNase III. Carrier state cultures exhibit a coordinate increase in the transcription of genes encoding ribosomal proteins that would also require an increase in RNase III to process rRNAs for the formation of ribosomes (Brathwaite et al., 2015). However, increased levels of RNase III in the carrier state cultures could lead to dysregulation of any regulatory RNAs that have the potential to fine tune regulation in a species that is noted for its minimal quota of regulatory genes. Non-coding RNAs CJnc10, CJnc60, CJnc160, and CJnc170 reported for *C. jejuni* NCTC11168 (Dugar et al., 2013) were conserved in *C. jejuni* PT14 but of these CJnc10 and CJnc170 RNAs were all but absent in the **carrier state** cultures. CJnc10 and CJnc170 were recently reported to be expressed from σ28 promoters and to effect translation of σ54-dependent transcripts through conserved complementary sequences in their 5′ untranslated regions (Le et al., 2015).

KEY CONCEPT 4 **Small RNA effectors of carrier state gene expression**

Antisense sRNAs have the potential to bind phage or host transcripts and act as *trans*-encoded inhibitory RNAs to suppress gene expression.

## Future perspectives

The consequences of expressing a phage-encoded Cas4-like protein in *C. jejuni* are intriguing and merit further study. The proposed role of Cas4-like protein as a mediator of the incorporation of host-derived CRISPR spacers would require controlled gene expression to suppress CRISPR-mediated autoimmunity. However, we note that Figure 2 contains alternative *Campylobacter* species in the second major clade that carry *cas4*-like coding sequences. These are also subject to modifications in genomic content, for example *Campylobacter fetus* subsp. *fetus* features a CRISPR array but this is partially deleted in *C. fetus* subsp. *venerealis* (Ali et al., 2012). The relationships between CRISPR-cas related functions of the genus will require further functional analysis.

The intricate relationship between *Campylobacters* and their associated bacteriophage has resulted in insurance mechanisms that allow for long-term replicative success of predator and prey. Short-term effects such as transient bacterial resistance to virulent phage infection following Mu prophage-driven genomic rearrangements may skew the replication bias toward the bacterium (Scott et al., 2007), albeit with a reduction in poultry colonization potential. However, restoration to phage sensitivity allows the wildtype population to recolonize poultry at the expected levels. In the carrier state virulent bacteriophage replication is accommodated whilst a subpopulation of bacteria remain resistant. The carrier state bacteria themselves are poor colonizers of poultry but are able to deliver bacteriophage to sensitive pre-colonized inhabitants (Siringan et al., 2014). Population level studies of bacteriophage/host interactions may shed yet further light on the nature of the success of *Campylobacter* as a major zoonotic foodborne pathogen.

## Author contributions

SH, KB, and IC reviewed experimental and computational data. SH and IC wrote the manuscript.

### Conflict of interest statement

The authors declare that the research was conducted in the absence of any commercial or financial relationships that could be construed as a potential conflict of interest.
